# Supplementary material for: Age-dependent electroencephalogram (EEG) patterns during sevoflurane general anesthesia in infants
Source: eLife. 2015 Jun 23;4:e06513. doi: 10.7554/eLife.06513 (PMC4502759; doi:10.7554/eLife.06513)
Supplement: Supplementary file 1. — Characteristics of individual infants. Subjects listed according to postnatal age. Data given for all infants included in the MOSSA analysis. M, months; MOSSA, Maintenance Of a Surgical State of Anesthesia; PNA, Postnatal Age; Wgt., weight. A–All infants were administered glycopyrrolate-neostigmine to reverse the neuromuscular blockade towards the end of surgery (except infants 1, 3, and 15). B–Infant was additionally administered clonidine for hypertension. DOI: http://dx.doi.org/10.7554/eLife.06513.018 [file elife06513s001.docx]

| No. | PNA (M) | Wgt  (kg) | Sex | Surgery | Nitrous oxide induction | Propofol (mg/kg) | Local anesthesia | Opioid analgesia | Neuromuscular blockade^A^ | Duration of anesthesia (min) | MOSSA  End-tidal sevoflurane (%) |
| --- | --- | --- | --- | --- | --- | --- | --- | --- | --- | --- | --- |
| 1 | 0.26 | 4.1 | F | Laparoscopic pull through |  | 10 | Ropivacaine | Fentanyl & morphine | Rocuronium | 646 | 0.8 |
| 2^^ | 0.53 | 3.7 | F | Anorectoplasty | - | - | Bupivacaine | Remifentanil | Rocuronium | 217 | 0.8 |
| 3 | 1.35 | 5.7 | M | Hernia repair |  | - | Bupivacaine | - | Rocuronium | 103 | 1.9 |
| 4 | 1.77 | 4.8 | M | Colostomy closure |  | - | Ropivacaine | Fentanyl | Rocuronium | 268 | 1.7 |
| 5 | 2.60 | 4.8 | M | Hernia repair |  | - | Bupivacaine | - | Rocuronium | 103 | 1.5 |
| 6 | 2.79 | 4.7 | M | Hernia repair & meatoplasty | - | - | Bupivacaine | Fentanyl | - | 114 | 2.6 |
| 7 | 2.83 | 5.0 | M | Hernia repair | - | - | Bupivacaine | - | Vecuronium | 79 | 2.3 |
| 8 | 2.89 | 6.3 | M | Hernia repair |  | 10 | Bupivacaine | Morphine | Rocuronium | 140 | 2.7 |
| 9 | 3.52 | 6.1 | F | Nephrostomy | - | 10 | Bupivacaine | Fentanyl | - | 170 | 2.2 |
| 10 | 3.58 | 5.7 | M | Hernia repair | - | - | Bupivacaine | - | Rocuronium | 118 | 2.0 |
| 11 | 3.81 | 7.6 | M | Fistula-in-ano excision |  | - | Bupivacaine | - | Rocuronium | 81 | 2.4 |
| 12 | 4.04 | 7.4 | M | Hernia repair |  | 10 | Bupivacaine | - | - | 94 | 2.6 |
| 13 | 4.80 | 6.3 | F | Hernia repair | - | 15 | Bupivacaine | - | - | 76 | 2.6 |
| 14 | 5.36 | 8.5 | M | Orchidopexy |  | - | Bupivacaine, lidocaine | Fentanyl & morphine | Rocuronium | 76 | 3.1 |
| 15 | 5.42 | 7.7 | M | Hypospadias repair |  | - | Bupivacaine | Morphine | Rocuronium | 144 | 2.2 |
| 16 | 5.49 | 8.2 | M | Lysis of penile skin tag |  | 20 | Bupivacaine | Fentanyl | - | 37 | 2.6 |
| 17 | 5.78 | 6.5 | M | Hypospadias repair | - | - | Bupivacaine | - | Rocuronium | 214 | 2.0 |
| 18 | 5.85 | 7.1 | M | Anoplasty |  | - | Bupivacaine, ropivacaine | Fentanyl | Rocuronium | 355 | 1.9 |
| 19 | 5.95 | 8.2 | M | Hypospadias repair |  | 20 | Bupivacaine, lidocaine | Fentanyl & morphine | - | 189 | 2.4 |
| 20 | 6.01 | 6.7 | M | Chordee release |  | - | Bupivacaine | - | Rocuronium | 160 | 3.4 |
| 21 | 6.05 | 8.4 | M | Circumcision |  | - | Bupivacaine | Fentanyl | - | 87 | 2.7 |
| 22 | 6.05 | 3.2 | M | Circumcision |  | - | Bupivacaine | - | - | 84 | 3.1 |
| 23 | 6.05 | 7.1 | M | Circumcision & penoplasty |  | - | Bupivacaine | Morphine | - | 94 | 2.5 |
| 24 | 6.08 | 9.0 | M | Orchidopexy |  | 10 | Bupivacaine | Morphine | - | 130 | 2.8 |
| 25 | 6.14 | 6.8 | M | Circumcision & hypospadias repair |  | 15 | Bupivacaine | Morphine | Cisatracurium | 145 | 2.8 |
| 26 | 6.14 | 7.9 | M | Chordee release |  | - | Bupivacaine | - | - | 66 | 2.6 |
| 27 | 6.28 | 7.8 | M | Circumcision & penoplasty |  | - | Bupivacaine | - | - | 74 | 3.4 |
| 28 | 6.51 | 8.2 | M | Circumcision |  | - | Bupivacaine | Morphine | - | 98 | 2.8 |
| 29 | 6.54 | 6.3 | M | Hypospadias repair |  | 15 | Bupivacaine, lidocaine | Morphine | - | 190 | 1.0 |
| 30 | 6.77 | 7.6 | F | Hernia repair |  | 15 | Bupivacaine | Morphine | - | 84 | 3.1 |

Supplementary File 1: Characteristics of individual infants. Subjects listed according to postnatal age. Data given for all infants included in the MOSSA analysis. M, months; MOSSA, Maintenance Of a Surgical State of Anesthesia; PNA, Post-Natal Age; Wgt., weight.

A-All infants were administered glycopyrrolate-neostigmine to reverse the neuromuscular blockade towards the end of surgery (except infants 1,3 and 15). B- Infant was additionally administered clonidine for hypertension.
